# Polyester-Based Coatings for Corrosion Protection

**DOI:** 10.3390/polym14163413

**Published:** 2022-08-21

**Authors:** Abesach M. Motlatle, Suprakas Sinha Ray, Vincent Ojijo, Manfred R. Scriba

**Affiliations:** 1 Centre for Nanostructures ad Advanced Materials, DSI-CSIR Nanotechnology Innovation Centre, Council for Scientific and Industrial Research, Pretoria 0001, South Africa; 2 Department of Chemical Science, University of Johannesburg, Johannesburg 2028, South Africa

**Keywords:** anticorrosion, polyester-based coatings, organic coatings, polymerization, bio-based coatings, cross-linking

## Abstract

The article is the first review encompassing the study and the applications of polyester-based coatings for the corrosion protection of steel. The impact of corrosion and the challenges encountered thus far and the solutions encountered in industry are addressed. Then, the use of polyesters as a promising alternative to current methods, such as phosphating, chromating, galvanization, and inhibitors, are highlighted. The classifications of polyesters and the network structure determine the overall applications and performance of the polymer. The review provides new trends in green chemistry and smart and bio-based polyester-based coatings. Finally, the different applications of polyesters are covered; specifically, the use of polyesters in surface coatings and for other industrial uses is discussed.

## 1. Introduction

Steel and iron are the most intensively used metals due to their affordability; however, they are susceptible to corrosion (rust), which is a global epidemic [1]. Corrosion is defined as the deterioration of metal from a chemical reaction prompted by environmental factors [2]. There are different types of corrosion, i.e., galvanic, stress, general, localized, intergranular, fretting, pitting, crevice, etc. [3]. Preventative methods have been reported, with unsatisfactory results. A corrosion cell consists of an anode, cathode, and an electrolyte solution; the electrolyte solution is in contact with the metal. Ionic species form at the anode and dissolve in the electrolyte solution [4]. Electrons flow from the anode to the cathode and a current is produced, which is used to determine the rate of corrosion of the metal. Different electrochemical reactions may occur on the surface of the metal, i.e., metal deposition, anodic, and cathodic reactions. In metal deposition, a metal is reduced from either a negatively or positively charged state to a neutral state [5]. An anodic reaction is when the metal reacts with the electrolyte from its neutral state and releases ions to form a corrosion current [6]. Lastly, in a cathodic reaction, the ions released through the anodic reaction are consumed by the cathode [7]. Iron (Fe) is the abundant component in steel, which is obtained by reduction of ores, such as haematite (Fe_2_O_3_), in a blast furnace with carbon. The reduction is written in simple chemical terms as follows:

2Fe_2_O_3_ + 3C → 4Fe + 3CO_2_  2.3 (iron ore) (coke) (iron) (↑gas)

High temperatures and energy are used in the process of manufacturing steel. Steel is unstable when exposed to moisture and oxygen and reverts to its original form in their presence: In simple chemical terms, the process is represented by;

Fe + O_2_ + H_2_O → Fe_2_O_3_.H_2_O  2.4 (iron) (oxygen) (water) (rust).

**Figure 1:**
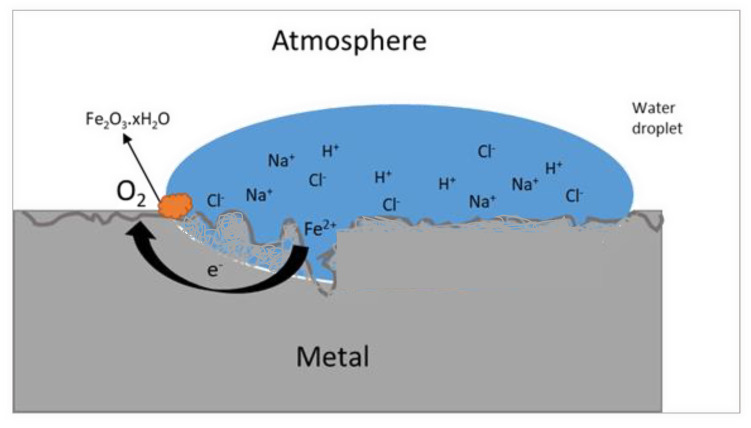
A depiction of the reaction between a coated steel substrate with a water droplet from the atmosphere.

Rust is a hydrated oxide of iron. However, the tendency for metals and metal oxides to revert to their original form can be explained by the thermodynamics of corrosion, which concerns the equilibrium state of a chemical system and the energy changes that occur [8]. Although thermodynamics provides information on the tendency of a reaction to occur, it provides no data on the rate of reaction or, in chemical terminology, the reaction kinetics [9]. It is known that steel, if exposed to moisture and oxygen, will rust as shown in the schematic diagram in Figure 1; however, in practice, the important question is usually how fast it will rust. 

**Figure 2:**
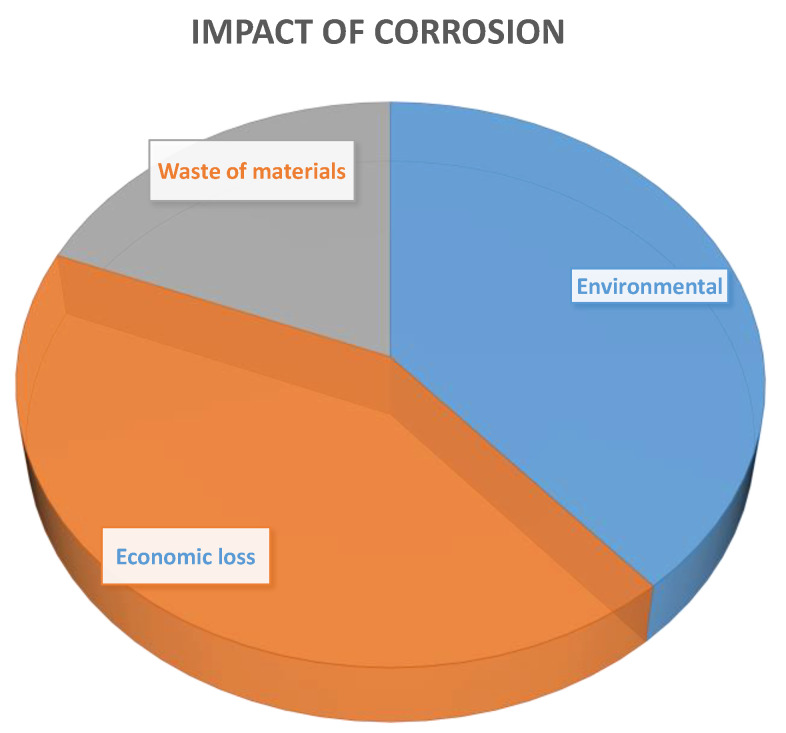
The impact of corrosion.

In South Africa, it is estimated that the cost of repairs, maintenance, and replacement of metal structures and equipment affected by corrosion is of the order of 4% of its gross domestic product (GDP) [10]. GDP is defined as the measure of global economic activity; it is used to monitor the spending on products and services rendered by consumers [11]. Researchers have had a huge interest in exploring the integration of materials by using new technology and science. However, corrosion remains a major economic problem [12]. Over the last years, the list of impacts attributed to corrosion have been extensively discussed. However, the waste of materials and environmental and economic losses are the fundamental effects resulting from corrosion due to their high cost implications and pollution. These effects contribute to the untimely failure of structures, which result in impairments to human and safety. Figure 2 indicates the proportion of results that are an impact of corrosion. Thus, there is a need for more research into and understanding of corrosion prevention and the development of cost-effective paint systems.

**Figure 3:**
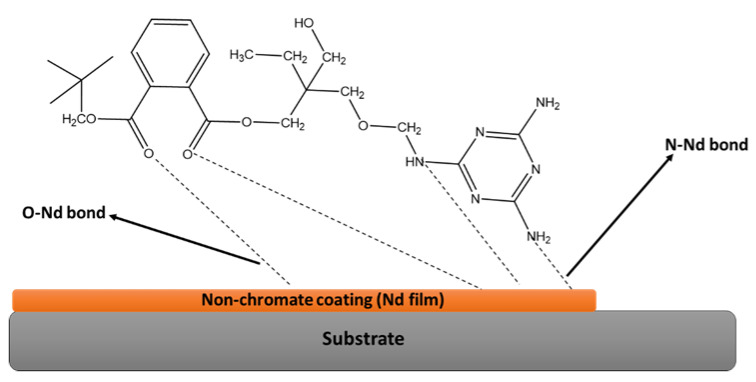
The significance of neodymium oxide film on the morphology, surface free energy calculations, and adhesion properties of a polyester/melamine coating [21].

Organic coating is the most common method used to inhibit or protect metal against corrosive mediums. However, there are environmental issues with the use of most coating systems, including epoxies, acrylic, polyurethanes, etc. Precautionary measures have been reported to have poor outcomes [13]. Surface coatings are modified films that form a barrier of protection between the film and the substrate. Reinforced polymers have enhanced properties when compared to their unmodified form; they can survive severe environments. However, the short-term protection offered by organic coatings is still costly, requiring the constant replacement of infrastructure as well as re-painting surfaces, hence there is a need for enduring alternatives. The current methods include galvanization of metals with zinc, phosphating, inhibitors, chromium-containing compounds (CCCs), and cathodic protection (Cp). The acidity level of rain influences the zinc corrosion rate in the outdoor environment for metallic coatings [14]. Phosphating processes are labor-intensive and render the work environment hazardous [15]. Cp results in poor adhesion properties while CCCs carry environmental and health concerns [16]. There still are drawbacks to the current coating systems. With the advances in technology bringing indispensable anticorrosion materials, coated metal-based materials have found wide applications in every aspect of life and all industries [17]. In brief, polyester-based coatings have attracted interest in recent years due to their affordability, good chemical resistance, low physical absorption, and stability [18]. Hasniraaiman et al. [19] investigated the effect of graphene dispersion in a polyester resin using mechanical stirring and sonication method for corrosion protection on carbon plate. The mixing technique is of great importance with carbon-conducting materials such as graphene. It was reported that a sonication method is better for the dispersion of graphene and influences the corrosion protection [20]. The ultra-sonication forces improve the exfoliation of the graphene sheets, resulting in superior corrosion protection. Many disadvantages remain for the current coating systems, which has led to the use of surface coatings. Bahlakeh et al. [21] studied the effect of a polyester and melamine coating based on neodymium oxide using experimental and molecular dynamics simulation, as seen in Figure 3 below. The researchers investigated the adhesion and anticorrosion efficacy when applied to steel substrates. The results showed increased adhesion strength, while the accelerated salt spray test and electrochemical techniques revealed good corrosion protection comparable to iron oxides. This study has proved that the use of harmful raw materials such as chromium is no longer the only option in obtaining coatings with strong surface properties and corrosion protection efficacy.

Polyesters are synthetic resins formed by an esterification chemical reaction with some occurring naturally [22]. In addition, there are different orientations of polyesters and, hence, different classifications. The classifications aid in determining the processing, curing kinetics, and overall applications of the resin [23]. Saturated, unsaturated polyesters (UPs) and alkyd resins are the main classifications of polyesters; however, vinyl esters are also classified as polyesters since they have a di-ester group. Vinyl esters are based on the combination of an epoxy resin with an unsaturated polymer; they have excellent properties when compared to saturated, unsaturated-type polyesters and alkyd resins [24]. The excellent properties include resistance to solvents, chemical and atmospheric attacks, as well as superior physical properties and corrosion protection [25].

However, the use of polyesters for corrosion protection has been neglected due to the high cost relative to the other polyesters as well as the short shelf life [26]. In this regard, the modification of polyesters with nanomaterials has been of interest to significantly improve the properties of surface coatings [27]. Developments of non-toxic polyester-based coatings have the potential to address a wide range of pollution problems, such as air pollution and water pollution, generated during the production of conventional polyester coatings [28]. The anticorrosion properties of polyester resin modified by nanocomposites intended for steel are of interest. The goal is to produce a bio-based polyester coating with minimal cost by implementing natural products as well as modifying with nanomaterials. The few review articles in the literature about polyesters investigate the synthesis and applications from a different point of view. There is particular emphasis on the curing kinetics [29,30,31,32], bio-based polyesters derived from renewable resources [33,34,35], and the thermal decomposition of polyesters [36,37,38,39]. The scope for this review is vinyl esters, their polymerization techniques, and the crosslinking processes; this is the first such inclusive study. In addition, the use of vinyl esters as surface coatings and the incorporation of nanomaterials for corrosion protection are described. The literature review section extends the current use of polyester-based coatings for corrosion protection and improves the properties of polyester coatings.

## 2. Polyesters

Polyesters are polymers formed from a dicarboxylic acid and a diol by a polycondensation process. Polyesters were first discovered by W.H. Carother while he was working for DuPont; however, his research was incomplete. In 1941, British scientists Whinfield and Dickson discovered the synthetic polyester fiber polyethylene terephthalate (PET) [40]. Polyester because popular in the 1970s due to its inexpensive and durable nature. Polyesters have various polymer backbones formed by esterification condensation; their applications depend on the parameters and the resulting orientation of the polymer chain [41]. The production of polyesters includes the addition of catalysts, promoters, curing agents, binders, solvents, and pigments. The characteristics of polyesters include strong fibers, mechanically durable, hydrophobic, retention of their original form, and easy to wash and dry. The drawbacks associated with polyesters are their low melting point, moisture absorption, toxicity, gelation during polycondensation reactions, and environmental and health hazards [42,43]. The global market for polyesters is set to increase by an average of more than 5% annually by 2020 due to the demand for and applications of polyesters [44].

**Figure 4:**
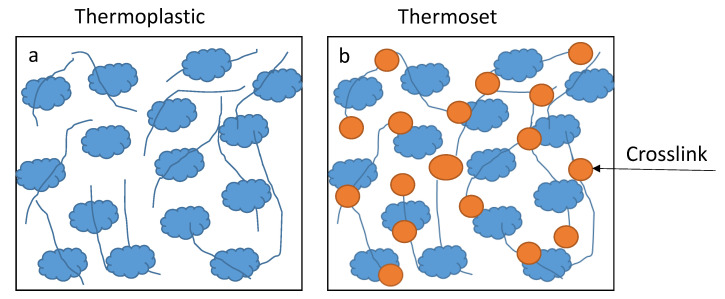
The crosslinking process between (**a**) weak intermolecular forces and (**b**) strong covalent bonds [46].

Polyesters can either be thermoset or thermoplastic polymers. Thermosets are formed by a compound with more functionality (covalent bonds) while thermoplastics are connected by weak intermolecular forces (see Figure 4) [45,46]. The rigid network structure formed by thermoset polymers gives rise to various applications due to the properties they possess [47]. Thermoplastic polyesters, PET and polybutylene terephthalate (PBT), are commercial products that are commonly used in the packaging industry [48]. Polyesters are classified by the orientation of the polymer chain as unsaturated, saturated polyesters, alkyd resins, and vinyl esters, respectively.

### 2.1. Unsaturated Polyesters

**Figure 5:**
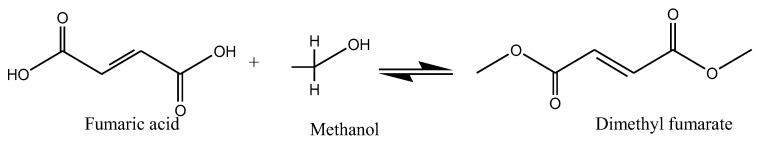
The chemical reaction between fumaric acid and methanol forms an unsaturated polyester resin (dimethyl fumarate) [53].

UPs are the largest group of polyesters. The backbone consist of alkyl thermosets resin [49]. The unsaturation in these polymers is due to a double or an olefin triple bond in the hydrocarbon chain of either the acid or alcohol. Poth et al. [50] defined UPs as building blocks of polycarboxylic acid-containing double bonds at the alpha position of carboxyl groups or their derivatives. UPs have been used since the 1930s due to their capability to be reinforced with other materials. Samsidin et al. [51] investigated an unsaturated polyester by modifying it with graphene grafted with silane at various weight percentages for application as a primer to carbon steel metal plates. The unsaturated polyester has poor mechanical and corrosion resistance when applied on its own, but adding a reinforcing agent to the polyester results in optimum performance of the primer coating. Graphene possesses good physicochemical properties; however, it has been reported in the literature that incorporation into polyester resin affects the polarity, which in turn affects the level of corrosion protection. Therefore, the researchers incorporated a silane coupling agent to compatibilize and improve the interaction of the polyester and graphene. The polymer was tested for corrosion using the immersion test and the Tafel test. It was discovered that the addition of 3% of saline solution to the polyester and graphene gives the lowest corrosion rate, of 0.148 mmpy, and the immersion test corroded area improved by 50%. A wide range of physical and chemical properties can be obtained from UPs depending on the application of the resin [52]. A chemical reaction illustrating the formation of UP resin from fumaric acid and methanol is presented in Figure 5; dimethyl fumarate is classified as an ester and used in the medical industry as an activator for some medications.

**Figure 6:**
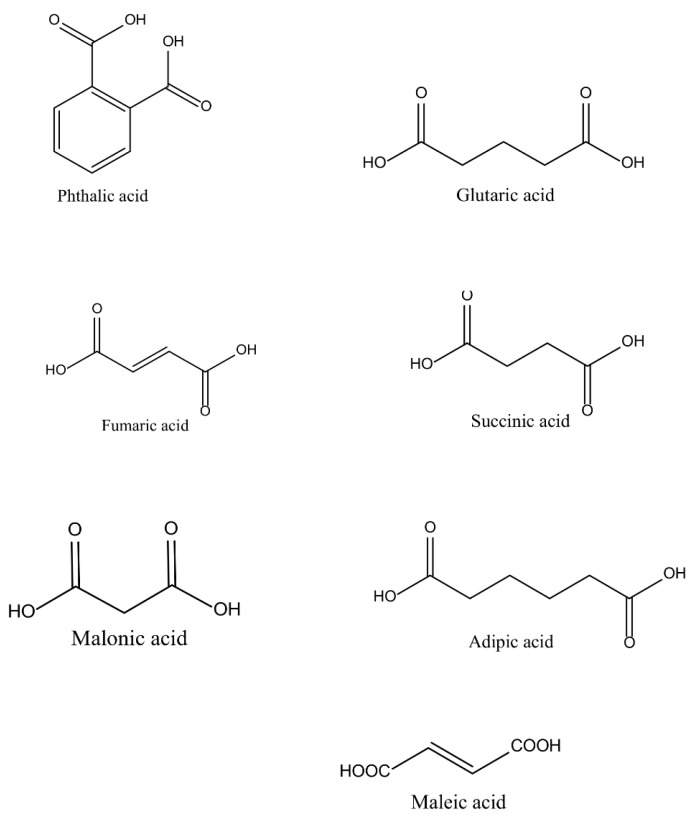
Some common chemical structures of acids that are used for the synthesis of UPs [59].

**Figure 7:**
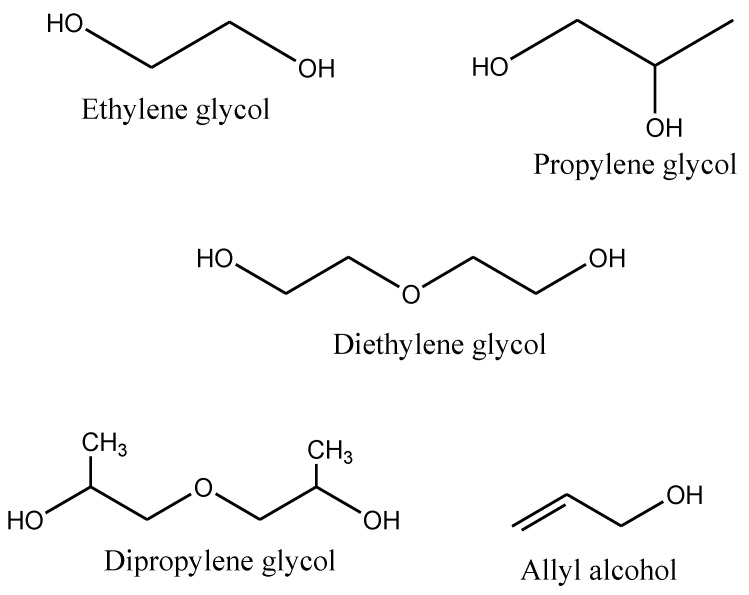
Some common chemical structures of alcohols used for the synthesis of UPs [60].

UPs are relatively inexpensive, mechanically strong, and possess tunable thermal properties. However, further modification of UPs properties with different fillers, additives, and pigments is a stimulating topic for advanced technology purposes [53]. The polymer can be reinforced with different inorganic and organic particles for better resistance to corrosion, weather, and flame-retardant properties [54]. The crosslinking of the polymers depends on the balance of the catalyst, inhibitors, and promoters [55]. In particular, the extent of cure depends on the materials used in the polymerization; the impenetrable structure is formed by crosslinking with oxygen, heat, and light [56]. The environmental aspect to reduce the amount of volatile used in UPs as crosslinkers and catalysts has been of great interest in attempts to minimize air pollution [57]. Li et al. [58] produced a self-curable UP, not by using crosslinking monomers but by adding vinyl groups on the end of the polymer chain to solve the problem of air pollution. The self-crosslinking reaction rate was seen to be greatly induced by the radical initiator (benzoyl peroxide) by increasing the polymerization reactivity attributed to the radical homopolymerization of the conjugated product. The raw materials commercially available for the production of UPs are petroleum based. The chemical structures of the acids and alcohols used in the synthesis of UPs are illustrated in Figure 6 and Figure 7, respectively. The acids include organic compounds, dicarboxylic acids, and aromatics; their applications are in the pharmaceutical, textile, and food industries [59]. The glycols and allyl alcohol are organic compounds used as raw materials in the production of polyesters and other pharmaceutical applications [60].

The interest in environmentally friendly UP resins has been extensively studied due to the worsening problem of air pollution. Bio-based UPs have been investigated to reduce the volatiles currently generated in the production of these polymers [61]. The use of renewable resources such as polymeric materials has been widely investigated due to the depletion of our fossil fuel reserves [62]. Dai and colleagues replaced petroleum-based with a bio-based UP 2,5-furandicarboxylic acid (FDCA) and itaconic acid. It was found to be thermally stable at 299 °C, with a 5% weight loss of the degradation temperature. The flexural strength increased from 116.8 to 122.8 Mpa with the inclusion of 6.8% FDCA in the polymer matrix. However, the glass transition temperature decreased from 141.7 to 127.6 °C, attributed to the crosslinking density induced by the double bond in the polymer chain. They concluded that bio-based UPs are comparable to, and even better than, petroleum-based UPs and can be applied instead [63]. Zhang et al. [64] reported the formulation of an environmentally friendly coil coating primer using a water-based saturated polyester. The effect of pretreatments, polymerizing agents, and the addition of fillers was investigated. Pretreatment is an essential part of coil coating of metal substrates, resulting in improved adhesion to the surface and overall good quality of the coated film. The type of additives used influences the quality of the overall coating; environmentally friendly pigments ZnMoO_4_, Zn_3_(PO_4_)_2_, AlH_2_P_3_O_10_, and Zn_3_Al(PO_4_)_3_ were instead of highly toxic chromates and lead-based pigments. The resulting coating showed excellent salt spray resistance at 360 h, good mechanical properties with 2H pencil hardness, and superior adhesion with minor improvements to the surface properties. Interest in the development of bio-based raw materials for coatings is increasing; however, cost implications and the availability of materials remain constraints.

### 2.2. Saturated Polyesters

**Figure 8:**
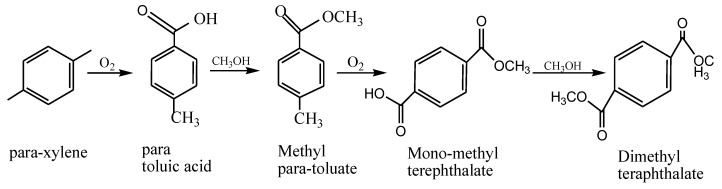
Reaction for the synthesis of a dibasic acid terephthalate.

Saturated polyesters are a type of polymer without hydroxyl groups resulting from the use of excess polyol or modifying the formulation [65]. Generally, a reaction between a dibasic acid with a diol will give a saturated polyester with an equivalent ratio poly-condensation process. They are linear and possess structures similar to simple polymers. Depending on the degree of polymerization, they are either solid or highly viscous [66]. The production of terephthalate follows the scheme in Figure 8.

**Figure 9:**
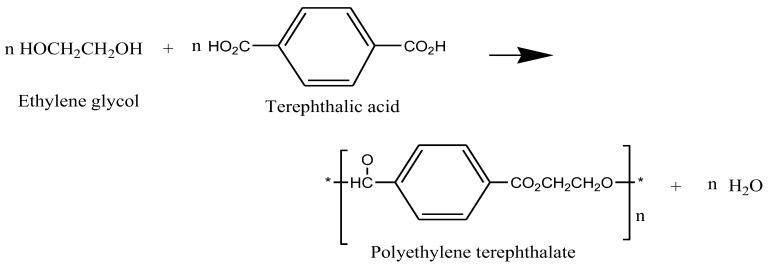
The chemical reaction of ethylene glycol with terephthalic acid to give a saturated polyester PET.

Terephthalic acid is a dibasic acid widely used for saturated polymers because of its chemistry. The esterification reaction is a condensation reaction initiated by the carboxyl group that can either be prepared by direct esterification, ester interchange process [67], or the reaction between a dihalide and an acid [68]. All the processes described form low-molecular-weight polymers, but these can be made into high-molecular-weight polymers by increasing the temperature. Other examples of saturated polyesters include poly(hexamethylene adipate), poly(ethylene adipate), di-isocyanate modified polyester, and poly (hexamethylene terephthalate). A condensation reaction between dimethyl terephthalate and ethylene glycol, giving a PET polymer, is illustrated in Figure 9.

Saturated polyesters have various properties, including high resistance to abrasion, bacteria, resistance to chemical attack, high strength, and flexibility, and possess good dielectric properties [69]. Their hydrophilic nature is responsible for the poor mechanical properties. Environments with high temperatures account for the decreased structural properties of saturated polyesters [70]. Lee and colleagues [71] investigated the synthesis of four types of flexible polyester resins with urethane polyol of polycarbonatediol (PCDL) isocyanate and carboxylic-terminated polyester pre-polymer. It was found that increasing the concentration of the polyol by approximately 300 ppm increased the flexibility of the polymer observed from the Fourier transform infrared (FTIR) spectrum detected at 1240 and 1760 cm^−1^ and the increased intensity of these bands. This was attributed to the soft segment and highly rubbery nature of the urethane polyol monomer. The polymer cures by free radical reactions, which are related to aging and oxidation. Dutta et al. [72] prepared a short oil polyester resin based on Mesua ferrea L. (Nahar) seed oil for stoving paint; the morphology and thermal stability were investigated. They found that the Nahar-based paint has excellent properties when compared to a castor oil-based stoving paint; however, the corrosion resistance was the same. They reported that this was due to the strong triazine and phenyl moieties as well as the three-dimensional (3D) network. Furthermore, research articles have been published based on saturated polyesters and their modification, as well as their use in anticorrosive coating [73,74].

### 2.3. Alkyd Resins

**Figure 10:**
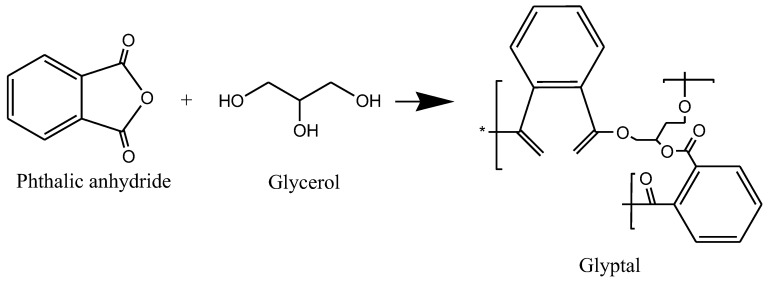
The reaction of glycerol with phthalic anhydride gives glyptal alkyd resin.

A reaction between a polyhydric alcohol and a fatty acid followed by a reaction with a dibasic acid gives an alkyd resin [75]. Glycerol is the most common type of polyalcohol used and phthalic anhydride is used as a polybasic acid. Alkyds can be modified by substituting a polybasic acid with a monobasic acid [76]. The polycondensation reaction between glycerol and phthalic anhydride giving polyester glyptal alkyd resin is illustrated in Figure 10.

Other polybasic acids used include maleic anhydride, fumaric acid, and isophthalic acid and the polyols used include ethylene glycol, trimethyloethane, and neopentyl glycol, among many others [77]. The properties of alkyds include compatibility with other coating polymers [78]. Alkyd resins have applications in paints, thermosetting polymers, and the printing industry [79].

### 2.4. Vinyl Esters

**Figure 11:**
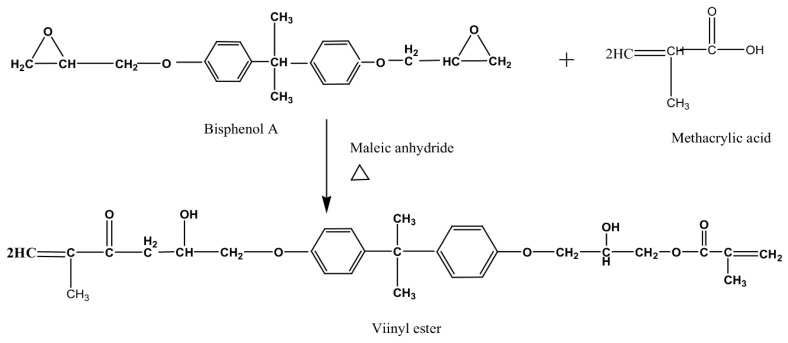
The reaction of bisphenol A and methacrylic acid with maleic anhydride as a catalyst at a temperature between 90–120 °C to form a vinyl ester resin.

Vinyl esters are polymers derived from vinyl alcohols [80]. The reaction of glycidyl acrylate and glycidyl methacrylate with bisphenol A through an esterification process is one example of forming the vinyl esters [81]. The functionalities are based on the combination of epoxy resin with an unsaturated polymer. There are very reactive with polar groups that give the excellent properties associated with vinyl esters. Vinyl esters are easy to handle at room temperature, which gives greater control over the curing rate [82]. However, the short shelf life is a drawback for quality control. A reaction to form a vinyl ester resin is illustrated in Figure 11.

Vinyl esters are sometimes classified as a polyester but they are di-esters due to the backbone of the chain that contains a link of ether groups [83]. The crosslinking is similar to that of UPs; however, the physical properties are superior due to the presence of olefin groups in the polymer chain [84]. The excellent properties of vinyl esters include good mechanical strength and resistance to chemical, solvent, and corrosion attacks [85]. Zhang et al. [86] studied the effect of isothermal temperature on the curing extent, gel time, rheology, and the mechanical properties of vinyl ester resin. The researchers found that the extent of cure depends on the isothermal temperature, while the tensile strength (higher elongation) increased with the increase in crosslinking density. The shear storage and loss modulus at the gel point were also observed to decrease with increases in both the isothermal temperature and the heating rate. The formation of micro-gels during the gelation process was the reason for this observation. 

The chemical and physical properties of vinyl esters are due to the size, the level of the polarity of terminal groups, arrangements of the monomers along the chain, and molecular weight [87]. Bio-based vinyl esters have been of most interest to improve the thermal and mechanical properties of vinyl esters. Winkler et al. [88] studied the thermal and mechanical properties of fatty acid starch esters (FASEs) by varying the degree of substitution of ester groups. The thermal stability was improved by more than 50 °C, which attributed to the starch obtained directly from plants while the mechanical properties decreased due to the supermolecular network of the polymer. It was concluded that the esterification process improves stability by reducing the rate of degradation of the starch. Vinyl esters tend to possess high molecular weights since nothing is eliminated during polymerization. Vinyl esters may be considered to be substituted ethylene due to their close relations to many polymeric coating and plastic resins, e.g., polyethylene, polystyrene (PS), polyvinyl chloride (PVC), and synthetic rubber [89]. Common resins commercially available in the market are listed in Table 1.

The different properties of vinyl ester resins are important for specific applications as shown in Table 1 above. The reactions to form the different commonly used vinyl esters are not the same, resulting in various reaction kinetics [91]. The polymerization rate is influenced by the resonance stability from the initiation step, the polarity of the olefins, and the steric hindrance [92]. The reactivity of vinyl esters has been enhanced by utilizing nanoparticles due to their high strength and high surface area [93]. Brand et al. [94] reported a novel method to tailor cellulose nanocrystal (CNC) using vinyl esters and found that the hydroxyl groups on the surface of the nanoparticles greatly influence the reaction efficiency as well as the kinetics. The influence was associated with either the kinetics being controlled by the diffusion of reactant and/or the catalyst within the CNCs agglomerates. The rate of reaction is influenced by the nucleophilicity of the surface on the hydroxyl groups. Anand and colleagues used glass fiber-reinforced composites at elevated temperatures to form a hybrid polymer composite (Ni-P coated glass fiber/Al_2_O_3_ nanowire-reinforced vinyl ester composite). The results showed improved storage modulus by 210% for the vinyl ester with 0.75 wt% Al_2_O_3_ nanowires and 44% Ni-P/GF as the reinforcement compared to glass fiber as the only reinforcement material. An increase in the start and end temperature from 450 to 505 °C, respectively, in the thermal stability of the reinforced composite [95]. Vinyl esters are formed by various methods according to the application, as previously mentioned; below is a more detailed discussion of the different polymerization reactions. 

#### 2.4.1. Polymerization of Vinyl Ester

There are four polymerization techniques for polymers: bulk, solvent, suspension, and emulsion processes. It has been mentioned that they are considered addition products of various epoxide resins and unsaturated acids. Many patents have been reported on the synthesis of vinyl esters ever since their discovery in the 1960s [96,97,98,99,100,101,102,103]. Oxidation is the degradation of the polymer during polymerization, which is a problem; however, it is minimized by the use of catalysts and reducing agents [104]. Polymerization at low temperatures yields polymers with a uniform backbone and high molecular weight, attributed to the slow reaction rates [105].

Primarily, bulk polymerization technique is a straightforward method of all four processes. The degree of polymerization in bulk polymerization is low due to the suspension of the monomer [106]. The processing is conscientious and requires a temperature control system. Some examples are PS, polyethylene, PVC, and polymethyl methacrylate. Secondly, solvent polymerization utilizes organic catalysts to form precipitation [107]. The polymer formed is expensive, tangible, and soluble in most organic solvents and has a high viscosity [108]. It is used for the production of polyvinyl alcohol (PVA), polyacrylonitrile, PVC, polyacrylic acid, polybutadiene, etc. Suspension polymerization is an aqueous process that forms stable colloids. Agitation is used to disperse the molecules to form a suspended solution, also referred to as bead polymerization [109]. Some examples include the production of PVC, polyvinyl acetate, PS, styrene-divinyl benzene, etc. Lastly, in emulsion polymerization, the monomer is emulsified in water. It is a commonly used method because of its ease of modification and the yield of high molecular weight polymers. Fast polymerization rates minimize production time, and the final products do not need further modification. Webster and his colleagues synthesized a cyclic carbonate functional polymer via the free radical solution copolymerization of vinyl ethylene carbonate with vinyl ester monomers [110]. They reported that the coatings produced had excellent solvent resistance to xylene, butyl acetate, dimethylformamide, propylene glycol monomethyl ether, methyl amyl ketone, ethyl ethoxypropionate, and propylene glycol monomethyl ether acetate. The solvent resistance was attributed to the amine crosslinkers that were utilized in the synthesis of the polymers. Production efficiency minimizes troubleshooting in the manufacturing process while also costing the company lots of money. Both the suspension and emulsion processes give resin that is less viscous and thermally stable; however, they are easily contaminated [111]. The crosslinking of vinyl esters resins is different from polyesters; however, all follow free radical polymerization. 

#### 2.4.2. Free Radical Polymerization

There are four steps involved in the free radical polymerization process: initiation, propagation, chain transfer, and termination. To start the polymerization, initiation is required to form a radical. Initiators are responsible for the crosslinking and the curing process of the resin [112]. A monomer reacts with a small amount of an initiator to produce a free radical. The radical formed in the initiation step reacts with the monomer to grow the polymer chain. The transfer of the radical can follow different pathways, either by a direct transfer to a monomer or another species or by the transfer of the radical to the solvent [113]. When the polymer has no growth, termination can then occur by two processes: coupling or disproportionation of the growing polymer. Free radicals are formed by several methods, including thermal decomposition and photochemical of peroxides azo compounds including their derivatives [114]. The polymer after the process of polymerization is then hardened, which is curing to a hard film, by adding catalyst as well as modifiers [115]. The vinyl ester chains form a 3D network structure that is crosslinked and this is the curing process.

#### 2.4.3. Crosslinking of Vinyl Esters

**Figure 12:**
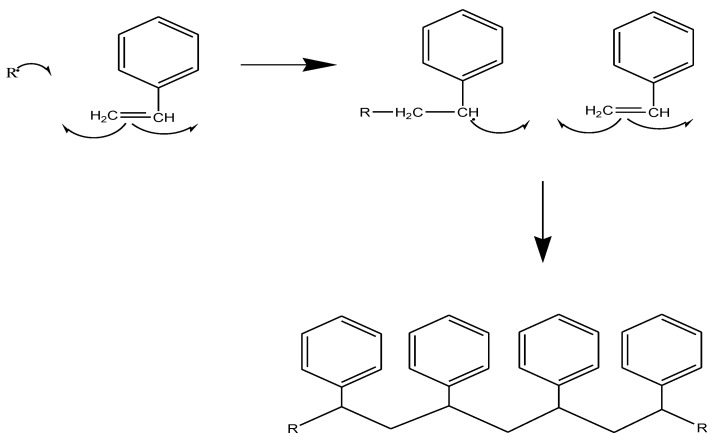
Styrene polymerization by free radical formation via electrophilic addition.

Vinyl esters follow the same mechanism as polyester by forming bonds with the side groups of a polymer chain. However, in this case, the polymerization of a vinyl ester is by free radicals, which initiates the crosslinking process. The initiator is responsible for the crosslinking of the resin; it can, however, be affected by heat, accelerators, and promoters in the polymerization. Ionization radiation is used to crosslink vinyl ester, which uses high energy, while electron radiation uses high quantum energy and forms the radical directly from the resin [116]. Ultraviolet radiation (UV) is also used with lower quantum energy by using photo-initiators to decompose the light and generate free radicals [117]. Radiation-curable coatings are promising because they are solvent-free, which is environmentally favorable [118]. An example of the crosslinking mechanism is illustrated in Figure 12.

**Figure 13:**
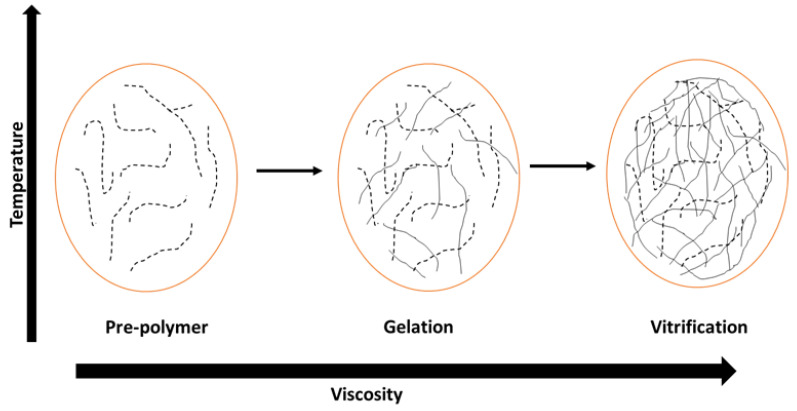
The cure diagram of a resin vitrification process [125].

The curing reactions of vinyl ester have been extensively studied and various kinetic models have been proposed [119,120,121]. Gelation and vitrification are the phenomena mostly investigated in the curing of vinyl esters. Gelation is defined as the time where a long polymer chain with high molecular weight is formed, whereas vitrification is defined as the temperature in which the glass transition (T_g_) is equal to the curing temperature of the resin [122,123]. The different phenomena can be represented in a time vs temperature transformation cure diagram. The three states (rubber, liquid, and glass) that occur are represented in Figure 13 above. The gelation, decomposition, and T_g_ of a polymer are important aspects that should be considered to obtain a product with satisfactory properties. The different states that occur determine the thermosetting properties of the polymer. The thermosetting properties determine the handling and processing due to the strong dependence on gelation and vitrification [124,125].

Differential scanning calorimetry has been utilized to study the kinetics of curing processes, which include the degree of cure and the time it takes to cure [126]. The polymerization and curing kinetics are mostly investigated with polyesters and vinyl esters, but not much has been reported on their corrosion protection on steel. Cook et al. [127] reported the curing kinetics of dimethacrylate-based vinyl ester resin and their thermal properties and found that temperature has an effect in the three different steps in a free radical polymerization. All the different classes of polyesters are applied in different industries due to their properties as well the network structure. Vinyl esters are neglected due to the high cost of the polymer resin and its production. The corrosion protection of vinyl esters has not been explored; thus, there is a need to review this phenomenon to understand the reason.

## 3. Developments in Polyester-Based Coatings

Similar work on polyester-based coatings and corrosion protection is outlined in this section. The articles and reviews on polyester-based coatings/resin as corrosion protection of steel and other metal substrates have been reviewed. Dai et al. [128] synthesized bio-based unsaturated polyester resins and their application in water-based coatings cured by UV radiation applied on a tin plate substrate. Three polyesters were formed with good properties and the reduction of the emission of volatile organic compounds was achieved in this production. Lee and his colleagues synthesized and characterized the polyester-based nanocomposites coatings for application in the automobile for pre-coated surfaces [129]. The polyethylene (PE) polymer was polymerized with various clay nanoparticles to form a composite; the pre-coated metal improve productivity. Good dispersion of the organically modified MMT in the PE polymer improved the mechanical, viscoelastic, and anticorrosion properties. The application using roll coating processes instead of a wet coating process decreased the problem of air pollution that occurs during evaporation. However, polyester-based compounds still have many drawbacks, such as the generation of volatile organic compounds, high labor intensity, emission of harmful by-products, and high costs. Atta et al. [130] studied unsaturated polyester resin based on rosin maleic anhydride adduct for the corrosion protection of steel. The findings of the study revealed that unsaturated polyesters based on the rosin adduct can be used for corrosion protection of steel with improved properties.

**Figure 14:**
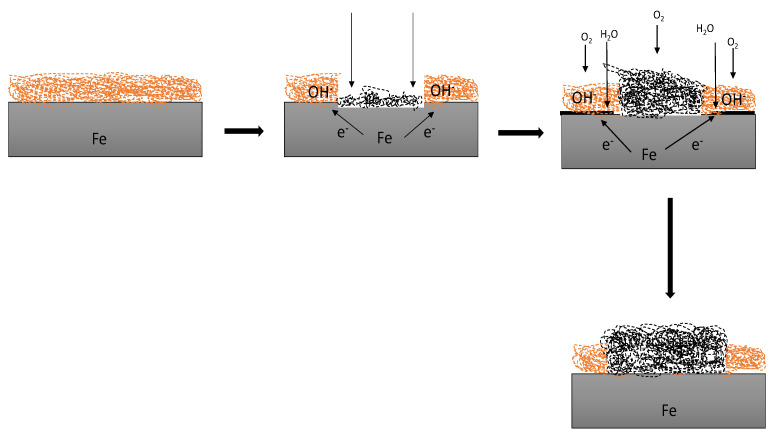
The degradation mechanism of a coated metal exposed to NaCl for 30 days from the initial state, when there was an attack from the atmosphere and the resultant product with rust.

Corrosion protection of carbon steel oil pipelines by unsaturated polyester/clay composite coatings was also investigated by Ramesh et al. [131]. The results indicated that the protected film surface stability and its resistance to dissolution were the crucial parameters in corrosion protection when applied to carbon steel. In a different study, Ramesh and his colleagues investigated silicone polyester blended coatings for corrosion protection. The hybrid system showed to have protection efficiency for more than 30 days, as well as good thermal properties. In references [132], the synthesis and characterization of hyper-branched polyester-urethane-urea (k10-clay) hybrid coatings was reported. The formation of 63% condensation product was achieved while the hydrophilicity of the hybrid coating is increased, attributed to the direct proportion increase in the clay and urea ratio. A similar study by Piazza et al. [133] showed that polyester-based powder coatings with montmorillonite (MMT) nanoparticles improved the anticorrosive properties when applied to carbon steel substrates. However, the thermal stability of the coating was decreased when higher concentrations of MMT were used, which was attributed to the oversaturation of the clay in the polymer matrix. Chen et al. [134] investigated the in situ polymerization and characterization of polyester-based polyurethane/nanosilica composite. It was reported that the chemical interaction in the polymerization improved the mechanical properties due to the nanoparticles being located in the interface and surface of the polymer when applied to tinned iron. The corrosion performance improvements of hot-dipped galvanized steel by electrodeposition of epoxy resin-ester modified BTSE (bis-tri-ethoxy-silyl-ethane) coatings were reported by Xue and colleagues [135]. They showed that coating using electrodeposition achieved better corrosion protection than immersion coating, which was attributed to the uniformity and non-porosity of the films. The application process of coating affects the overall performance of the coating. The excellent properties of polyesters will reduce the need for re-application processes and replacement of damaged structures affected by corrosion as depicted in Figure 14.

Vinyl esters are mostly used for excellent mechanical and adherent properties and resistance to corrosion attack [136]. However, the short shelf life is a drawback for quality control. Gopi et al. [137] synthesized and characterized the corrosion protection of poly (N-vinyl carbazole-co-glycidyl methacrylate) coatings applied on low nickel stainless steel. The results showed that the ratio of the copolymer improved the corrosion protection in 0.5 M H_2_SO_4_ medium by the formation of a barrier effect from the polymer layer. The protection efficiency is highly dependent on the composition of the copolymer. In another study, Hollamby and colleagues investigated the hybrid polyester coating incorporating functionalized mesoporous carries for the holistic protection of galvanized steel surfaces [138]. A 96% corrosion efficiency was achieved, attributed to the use of a dense polymer and mesoporous silica nanoparticles.

Ali et al. [139] developed a new polyester acrylate resin from palm oil for wood applications. Trimethylolpropane triacrylate (TMPTA) and 1,6-hexanediol diacrylate (HDDA) proved to be more suitable diluents with palm oil, producing a stable coating. The use of renewable resource materials such as palm oil makes the coating an environmentally friendly option with the UV radiation-curable feature saving energy. The process to develop new, innovative ways of polyester production has increasingly becoming the focus of the paint industry. This is seen by researchers such as Abbate et al. [140] reporting on a novel reactive liquid rubber with maleimide end groups for the toughening of the unsaturated polyester resins. The amino-terminated butadiene–acrylonitrile copolymer showed higher toughness at low concentrations when compared to the unmodified liquid rubber. The modification of polymers with nanoparticles or macro particles has been studied and proved to highly improve the efficacy and overall performance of the polymer matrix. Marian et al. [141] investigated the thermal properties of polyester/graphene oxide and graphite by thermomechanical analysis. Graphite was found to have an insignificant influence on the thermal properties than graphene oxide attributed to the coefficient of linear thermal expansion (CLTE). The use of polyvinyl chloride (PVC)-based plastisols emits fumes such as phthalates, which encouraged the paint industry to focus its research on producing paint formulations with additives for improved corrosion protection on metal surfaces that were environmentally friendly [142]. However, through the incorporation of nanoparticles and nanostructures, a paint can, for instance, be made conductive [143], more scratch resistant [144], compatible with surfaces, and more resistant to fire and heat [145]. These improvements are partly due to the advantage conferred by nanoparticles having significantly larger surface areas than ordinary micron-sized particles, which increases chemical reactivity [146]. Many disadvantages remain for the current coating systems; hence, protective surface coatings are used.

## 4. Applications

**Figure 15:**
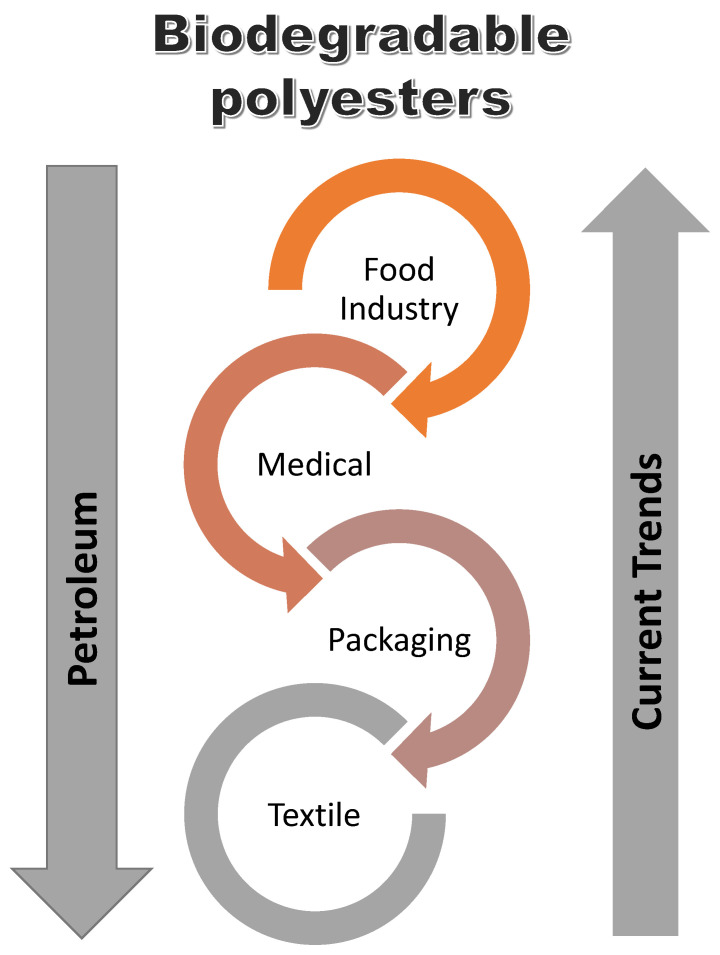
Current trends in the use of biodegradable polyester vs petroleum-based coatings [147].

The applications of polyesters and vinyl esters depend on the properties of the resin. Different industries apply polyester and vinyl ester resin due to their excellent properties as already discussed. The different industries are outlined in Table 2 and the potential growth in the application of biodegradable polymers is depicted in Figure 15.

## 5. Conclusions

The development of polyester-based resins has primarily been explored in the polymer packaging industry. The thermal, kinetic, and flame-retardant properties of polyester resin are mainly considered. Different synthesis methods have been developed for polyester resins with excellent properties, resulting in the publication of many patents. There is still a need to reduce the energy consumption in the coating industry to reduce carbon dioxide emissions and production costs. The problem of volatile organic compounds (VOCs) is the reason why there are limitations in the use of these polymers. Powder coatings have been mostly used instead of solvent-borne coatings to reduce the use of VOCs. The conversion of biomass to the synthesis of bio-based polyesters has been explored as a means to prevent the depletion of natural resources. Blending polyesters with other polymers has been largely attempted to overcome the limitations of the polymers and enhance the properties of the end functional polymer, such as corrosion protection. Corrosion protection has been investigated by using blends instead of neat polyester polymers. The curing of polyester remains a highly investigated topic for this group of polymers. Hyper-branched polyesters have been explored for their hydrophilicity in coatings. Untreated steel and mild steel substrates are seldom used for the application of polyester coatings. The corrosion protection of polyester coatings on steel is not reported because of the high curing temperatures needed. Vinyl esters are mostly used for excellent mechanical and adherent properties and resistance to corrosion attack. The production of vinyl esters compared to polyester coating will be costly, but the excellent properties will reduce the frequency of the re-application process and replacement of damaged structures. There are a few review articles focusing on polyesters; however, there is still a need for more research that presents an overview of polyesters and vinyl esters coatings for corrosion protection from different points of view.

## Figures and Tables

**Table 1:** Common resins on the market [90].

Resin	Structure	Polymer	Properties	Application
Styrene	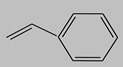	Polystyrene	Transparency, water-resistance, and excellent electrical property	Injection molding
Vinyl chloride	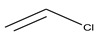	Polyvinyl chloride	Unique elasticity, fatigue, and water resistance	Synthetic textile
Vinyl acetate	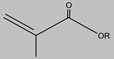	Polyvinyl acetate	Light and heat stability	Adhesives, dipping lacquer for artificial leather and synthetic fibers
Acrylic acid	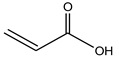	Polyvinyl	Soluble in various solvents, transparent	Disposable diapers, resin, and adhesives
Methacrylic acid esters	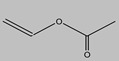	Polymethacrylates	Strong, light, and good impact strength	Automotive, appliances, and glasses lenses

**Table 2:** Other applications of polyesters.

Industry	Type of Polyester	Properties	Reference
**Transportation**	Polybutylene-terephthalate (PBT) Poly (ether sulfones) (PES)	High resistance to temperature High strength Hardness Excellent sliding properties Electric insulation High resistance to abrasion and modulus	[148,149,150]
**Electrical**	Polypropylene (BOPP)	Dry without losing volatiles Excellent mechanical strength	[151,152,153]
**Industrial applications**	Polyvinylidene fluoride (PVDF) Fluorinated ethylene propylene (FEP)	Chemical resistance Good corrosion properties Longer shelf life prolongs the lifespan of the equipment	[154,155,156,157,158]
**Construction**	Ethylene tetrafluoroethylene (ETFE)	Elasticity Structure with lower weight Resistance to thermal changes Mechanically strong Chemical resistance	[159,160]

## Data Availability

Supplementary data is available upon request.
